# Hand osteomyelitis in arterial calcification, diabetes mellitus and end-stage renal failure: a comparison of 210 cases over 12 years

**DOI:** 10.1177/1753193420981871

**Published:** 2021-01-17

**Authors:** Matthew Wyman, Dallan Dargan, Jennifer Caddick, Victoria Giblin

**Affiliations:** 1 Sheffield Teaching Hospitals NHS Foundation Trust, Northern General Hospital, Sheffield, UK; 2 Academic Medical Unit, The University of Sheffield, Sheffield, UK

**Keywords:** Osteomyelitis, hand, vascular calcification, ischaemia, peripheral arterial disease, debridement, amputation

## Abstract

We present 210 patients with hand osteomyelitis in 246 rays over 12 years, including detailed analysis of 29 patients in this cohort with digital artery calcification evident on plain X-ray. Overall 71 patients had diabetes mellitus and/or end-stage renal failure, including 28 of 29 patients with calcification. In the calcification group, 17 patients had ipsilateral arteriovenous fistulae, five had steal syndrome and 15 had digital ulceration or skin necrosis. Compared with 181 controls, patients with calcification had more affected bones, polymicrobial infections, surgical procedures, phalanges and digits amputated and had higher mortality at 1 year (12 of 29) and 5 years (20 of 29), as a result of comorbidities. Absence of calcification in 43 patients with diabetes and/or end-stage renal failure was associated with better outcomes on all the above parameters. Early amputation to maximize disease-free survival may be appropriate for patients with hand osteomyelitis and arterial calcification.

**Level of evidence:** IV

## Introduction

Hand osteomyelitis is relatively uncommon, comprising 8% of osteomyelitis cases, whereas feet are affected in 43% of cases (Kremers et al., 2014). An antecedent can often be identified, such as a bite or injury. Surgical debridement may be required, with loss of functional finger length or amputation.

The diagnostic challenges with long bone osteomyelitis are well documented (Fritz and McDonald, 2008; Hatzenbuehler and Pulling, 2011), and guidelines (Public Health England, 2016) suggest a combination of clinical, imaging, biopsy and surgical assessments. Fracture-related infection guidelines (British Orthopaedic Association, 2019) advocate seven bone samples per patient, which could potentially destroy a phalanx. Reilly et al. (1997) reported 46 patients with hand osteomyelitis and identified an amputation incidence of 39% (18 of 48 hands). Henry and Lundy (2019) described a low-cost oral antibiotic protocol with better outcomes for 69 patients with hand osteomyelitis.

Cierny and Mader (2003) recognized that comorbidities are prognostic indicators for osteomyelitis. Peripheral vascular disease (PVD), which is prevalent in diabetes mellitus (DM) and end-stage renal failure, increases the incidence of infection and amputation in feet (Huang et al., 2014; Johnson et al., 2019) and overall morbidity and mortality (Adragao et al., 2004; Snell-Bergeon et al., 2013). Arterial calcification severity is categorized using the Agatston method (Agatston et al., 1990). This assesses the total of calcified lesions with a cross sectional area of >1 mm^2^ and density of >130 Hounsfield units on CT, and high scores in lower limb PVD are strongly associated with amputation after adjustment for risk factors (Guzman et al., 2008; Huang et al., 2014). Hand X-ray arterial calcification is prevalent in rheumatoid arthritis (99 of 906 patients, 11%) and has an increased all-cause mortality (Blair Solow et al., 2015).

The purpose of this study was to compare patterns in aetiology, comorbidities and outcomes in patients with hand osteomyelitis, with and without digital artery calcification and to establish whether this radiological finding confers useful diagnostic, prognostic or therapeutic information.

## Methods

### Caseload identification

This retrospective observational cohort study at a major trauma centre was approved by the institutional review board, study number STH20214. A multifaceted approach was taken to generate the study population. Imaging reports of hand or fingers (X-rays, CT, magnetic resonance imaging (MRI), ultrasound) from 2008 to 2019 inclusive containing keywords ‘osteomyelitis’ or ‘septic arthritis’ were retrieved. Theatre management software was interrogated for amputations and bone biopsies of metacarpals and hand phalanges performed during the same period, and daily trauma handover lists from 2016 to 2019 inclusive were searched for osteomyelitis cases. A total of 2464 records were screened, of which 341 had evidence of hand osteomyelitis and were selected for further review.

### Diagnosis of osteomyelitis and arterial calcification

Osteomyelitis was diagnosed using a combination of microbiological sampling, clinical findings and imaging. Bone biopsy was the preferred method for microbiological confirmation; alternatively, clinical findings (gross purulence, a discharging sinus over bone or clinical soft tissue infection with exposed bone in combination with positive radiographic findings) was diagnostic, where other causes had been excluded. Carpal osteomyelitis was excluded. Of the 341 patients reviewed, 210 met these criteria and were confirmed to have hand osteomyelitis. Hand and finger X-rays of the population with confirmed hand osteomyelitis were assessed for the presence of arterial calcification by the first author. A sample was independently reviewed by a second, blinded researcher, and all calcification-positive X-rays were reviewed by a consultant radiologist. Non-arterial soft tissue calcification (in scleroderma), and arterial calcification on imaging techniques other than plain X-rays, was disregarded.

### Hand osteomyelitis cohorts and definitions

Individuals with calcification of any digital artery or of the deep or superficial palmar arch on an ipsilateral hand or finger X-ray prior to diagnosis of osteomyelitis were included in Cohort A (artery calcification), regardless of comorbidities. Individuals without calcification were divided into two control cohorts: those with DM or end-stage renal failure comprised Cohort B, and all others were included in Cohort C. Outcomes for each cohort were compared. Follow up was until discharge from surgical and hand therapy review, and our centre was routinely notified of the death of any current or past patients. Involvement of a ray was defined as involvement of any phalanx or metacarpal of that digit.

### Statistical analysis

Normally distributed variables were described as means, standard deviations (SD) and ranges. Binomial data were compared using risk ratio (RR), 95% confidence intervals and Pearson’s chi-squared test or Fisher’s exact test for values < 5. Non-parametric data were described as median, interquartile range and range, compared using the Mann–Whitney *U* test. Survival analyses were represented as Kaplan–Meier plots, and adjacent survival curves were compared using the log rank test.

## Results

Hand osteomyelitis occurred in 246 rays of 210 individuals. A total of 130 individuals underwent bone biopsy (62%). Histological confirmation of osteomyelitis was available in 32 individuals (15%). Ten patients were intravenous drug users. Ten had secondary Raynaud’s phenomenon, of which seven were analysed separately (Cox and Hughes, 2020; Haque et al., in press). Neither group had arterial calcification or sickle cell anaemia. A history of smoking was similar in Cohorts A, B and C (48%, 37%, 43%), respectively. Mean age was similar between cohorts (A 62.5 years, SD 13.5; B 65.0 years, SD 13.9; C 54.6 years, 18.0). Analysis of microorganisms and inflammatory markers is being undertaken separately.

### Cohort A

**Figure 1.**
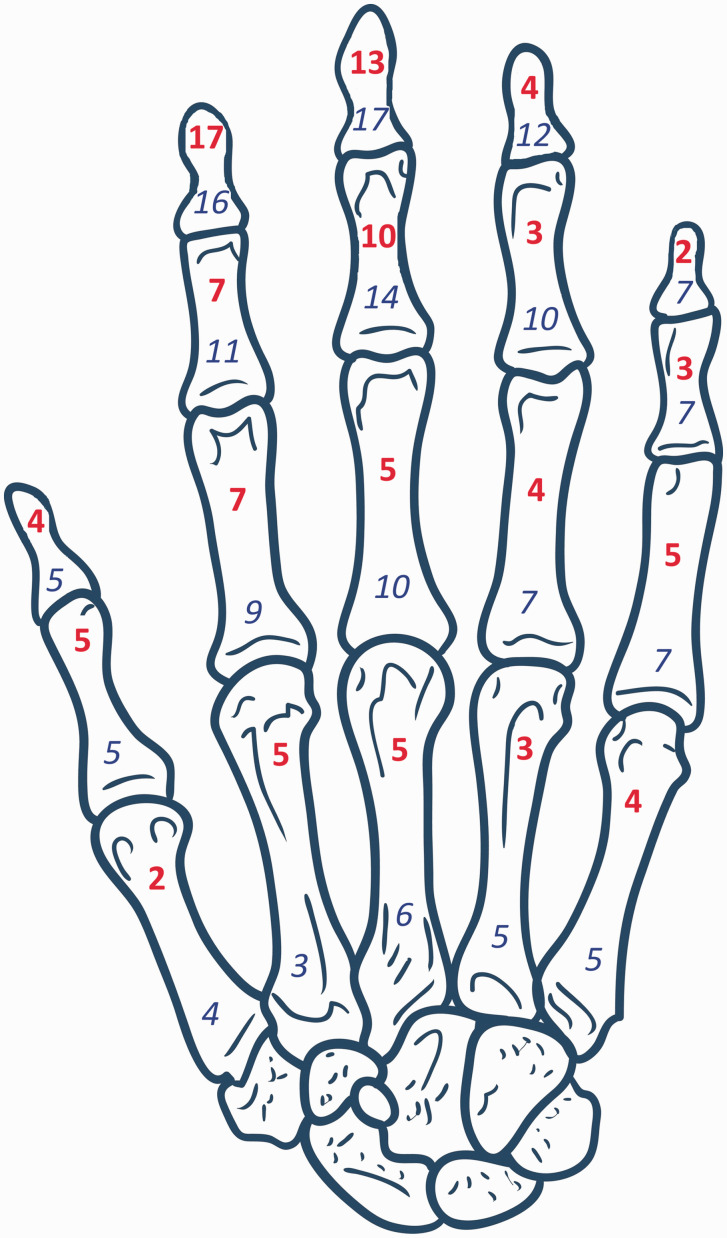
Incidence of osteomyelitis (in red, bold font) and frequency of amputation (in blue, italic font) of individual phalanges and metacarpals at presentation in a cohort of 29 patients with osteomyelitis and arterial calcification (Cohort A). Original hand bone diagram used in Figure 1 created by © www.gograph.com / Chuhail. Permission granted for use and adaptation.

**Figure 2.**
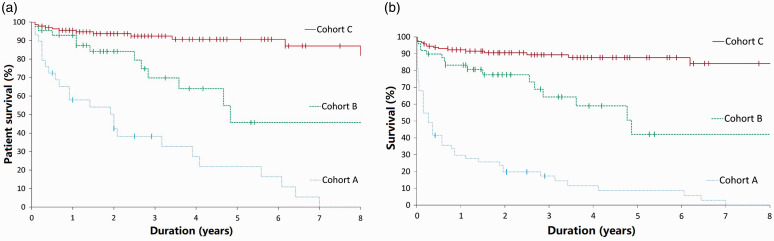
Kaplan–Meier plots for three cohorts of hand osteomyelitis: A – digital arterial calcification; B – DM or end-stage renal failure without arterial calcification; C – all others. Log rank test Cohort A versus Cohort B, *p* < 0.001, and Cohort A versus Cohort C, *p* < 0.001 in both graphs. (a) Overall patient survival, indicating high incidences of mortality from comorbidities, with no participant in Cohort A surviving beyond 7 years from diagnosis of hand osteomyelitis with arterial calcification. Dashes indicate surviving patients and the duration of their survival from diagnosis of hand osteomyelitis. (b) Amputation-free survival (excludes partial or phalanx amputations) from time of diagnosis, indicating a poor prognosis and high digit or ray amputation rate for patients in Cohort A. Dashes indicate surviving patients who did not require complete amputation of any digit and the duration of their survival from the diagnosis of hand osteomyelitis.

**Figure 3.**
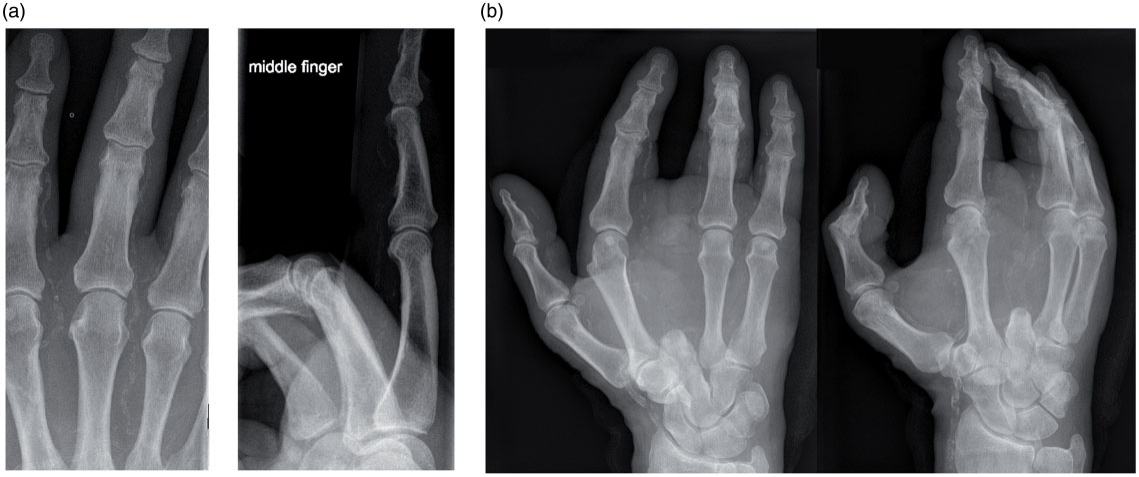
(a) Posteroanterior and lateral X-rays of a right middle finger from a 72-year-old man with arterial calcification taken at the time of diagnosis of osteomyelitis. (b) Posteroanterior and oblique X-rays taken 5 months later following phalanx amputation of the right middle finger at the proximal interphalangeal joint 1 month after diagnosis and then complete ray amputation a month later.

In Cohort A (29 patients with 53 affected rays), end-stage renal failure was comorbid in 20 patients (69%), DM in 25 (86%) and both concomitantly in 17 patients (59%). One patient had neither comorbidity. Twenty-seven of 29 patients were male (93%). At diagnosis, 16 patients (55%) had undergone transtibial, knee disarticulation or transfemoral amputations; a further three (10%) had undergone toe amputation(s). The aetiology was ischaemic ulceration or skin necrosis in 15 patients or a trivial injury with poor wound healing (13 patients). At least 11 reported paraesthesia or ischaemic rest pain in the weeks preceding diagnosis. Sixteen patients (55%) underwent formal vascular imaging, such as a CT angiogram, MR angiogram, arterial ultrasound or fistulogram, of the affected arm. Complete occlusion of either the radial or ulnar arteries was noted in four, and angioplasty was performed in two. Among 18 with arteriovenous fistulae (62%), osteomyelitis was ipsilateral in 12, bilateral in five and contralateral one; steal syndrome was documented in five. The distribution of bones involved and amputated in Cohort A is depicted in Figure 1. Twenty-four patients (83%) in Cohort A had died by study completion (range 1 day to 7 years from osteomyelitis diagnosis). From the time of the diagnosis of osteomyelitis, all-cause mortality at 5 years was 69% (20 of 29, Figure 2(a)). At 1 year from diagnosis, 37 of the 53 affected digits (70%) had been amputated completely, or the patient had died. The median time from diagnosis to complete amputation (or death) was 4.4 months (Figure 2(b)). An example of digital artery calcification and subsequent ray amputation following a series of partial amputations is presented in Figure 3(a) and (b).

### Cohort B

Forty-nine rays among 43 individuals were affected in Cohort B. Diabetes mellitus was present in 41 (95%), and end-stage renal failure in 2 (5%); none had both concomitantly. Twenty-eight of 43 patients were male (70%). The aetiology was trauma in 11 patients (26%) and contiguous abscess (paronychia, pulp space abscess, flexor sheath infection) in nine patients (21%).

### Cohort C

Cohort C comprised 138 individuals with 145 affected rays. Eighty-four of 138 patients were male (61%). The most common aetiology was trauma in 58 patients (42%), followed by contiguous abscess in 29 patients (21%), although aetiologies overlapped in some.

### Comparison of three cohorts

Compared with the 181 patients without arterial calcification, the 29 with calcification were more likely to have multiple bones affected by osteomyelitis (79% versus 34%), have a higher rate of polymicrobial infection (20 (69%) versus 65 (39%)), require multiple procedures more often (62% versus 29%), require more digit/ray amputations (12 (41%) versus 9 (5%)) and have higher all-cause mortality at 1 year (12 (41%) versus 9 (5%)). All of these differences achieved statistical significance (Table 1).

**Table 1.** Outcomes for 210 patients with hand osteomyelitis, represented as Cohorts A, B and C.

Outcomes	Cohort A Arterial calcification, 29 patients	Cohort B DM and/or ESRF but no calcification, 43 patients	A vs B RR (95% CI) *p*-value	Cohort C All others, 138 patients	A vs C RR (95% CI) *p*-value
Multiple bones involved (%)	23 (79%)	19 (44%)	1.8 (1.6–14.3) *p* = 0.003, χ^2^	49 (36%)	2.2 (2.7–18.3) *p* < 0.001, χ^2^
Polymicrobial infections (%)	20 (69%)	19 (44%)	1.6 (1.0–7.6) *p* = 0.038, χ^2^	46 (33%)	2.1 (1.9–10.5) *p* < 0.001, χ^2^
Multiple procedures required (%)	18 (62%)	12 (28%)	2.2 (1.5–11.5) *p* = 0.004, χ^2^	40 (29%)	2.1 (1.7–9.2) *p* < 0.001, χ^2^
Phalanx amputation (%)	25 (86%)	17 (40%)	2.2 (2.8–32.4) *p* < 0.001, FE	40 (29%)	3.0 (5.0–46.8) *p* < 0.001, FE
Digit/ray amputation (%)	12 (41%)	2 (5%)	8.9 (2.9–71.7) *p* < 0.001, FE	7 (5%)	8.2 (4.6–38.1) *p* < 0.001, FE
1 year mortality (%)	12 (41%)	3 (7%)	5.9 (2.4–37.7) *p* < 0.001, χ^2^	6 (4%)	9.5 (5.2–46.8) *p* < 0.001, χ^2^
Treatment duration, days^a^	80 (178, 1–1781)	53 (78, 1–393)	*p* = 0.148	71 (105, 1–836)	*p* = 0.367
Cumulative inpatient stay, days^a^	17 (24, 1–234)	3 (6, 0–53)	*p* < 0.001	2.5 (4, 0–50)	*p* < 0.001

RR: risk ratio; CI: confidence interval; SD: standard deviation; DM: diabetes mellitus; ESRF: end stage renal failure; χ^2^: chi-squared test; FE: Fisher’s exact test.

a Non-parametric data, presented in the table as median (interquartile range, range) and statistical comparison using Mann–Whitney *U* test.

## Discussion

Arterial calcification in hand osteomyelitis predisposed to multiple surgeries and digit shortening in our series. Calcification was present in 14% of our patients, similar to the 11% prevalence found in a series of hands with rheumatoid arthritis (Blair Solow et al., 2015). Our arterial calcification cohort consisted almost exclusively of patients with DM and/or end-stage renal failure (97%). These conditions are associated with lower extremity osteomyelitis and high incidences of amputation ranging from 36% to 95% (Aragón-Sánchez et al., 2008; Bahebeck et al., 2010; Xu et al., 2010). Similarly, we found 86% of diabetic patients with end-stage renal failure in our series lost one or more phalanges. In the calcification cohort, poor wound healing often resulted in progressive infection and surgical shortening of the same digit over days or weeks, reminiscent of critical limb ischaemia in PVD (Slovut and Sullivan, 2008; Yeong et al., 2019). Nineteen of 29 (66%) had prior lower limb amputation and PVD, and approximately one-third reported hand paraesthesia or ischaemic rest pain.

The poor prognosis with arterial calcification in our series suggests that early, aggressive debridement or amputation may be optimal. Conversely, two frail and comorbid patients with arterial calcification were categorized as Cierny–Mader host status C (treatment worse than the disease) and did not undergo debridement or amputation. This suggests that the Cierny–Mader host classification remains relevant for identifying patients who are not surgical candidates (Cierny et al., 2003).

Five patients in our series had radiologically evident steal syndrome, which is associated with tissue necrosis; however, others may have had clinical evidence of steal, such as hand pain, especially during dialysis. Fistulae were usually sited in the non-dominant hand, and osteomyelitis in the left hand was more extensive and prevalent. Detected early, tissue loss from steal syndrome can by minimized but not halted by reducing diastolic inflow into the fistula through banding or revascularization; these techniques, however, are often impossible with extensive vascular calcification (Mickley, 2007). Patients with steal syndrome often presented late in our centre after irreversible tissue loss has already occurred. Tissue death from steal syndrome can be reduced through fistula ligation, in which case alternative fistula formation is necessary for haemodialysis.

This retrospective study and the challenge of defining osteomyelitis present some limitations. Histological bone sample confirmation was low (15%). Hand digital arterial calcification manifests only in severe PVD (Adragao et al., 2004), producing selection bias for severe disease. The collinearity between arterial calcification and DM, PVD and end-stage renal failure confounds interpretation of the high amputation and mortality rates observed. Bone destruction may be primarily ischaemic, rather than infective, with subsequent microbial colonization. X-ray imaging is not comparable with upper extremity vascular contrast imaging or arterial ultrasound but is rapid, non-invasive and inexpensive. Identifying arterial calcification is prognostic in hand osteomyelitis and warrants consideration of clinical and radiological assessment for PVD (National Institute for Clinical Excellence, 2012).

Patients with hand osteomyelitis and arterial calcification evident on plain X-rays have more bones involved, higher incidences of polymicrobial infections and amputations, and higher 1-year and 5-year mortalities as a result of comorbidities.
